# NOA36 Protein Contains a Highly Conserved Nucleolar Localization Signal Capable of Directing Functional Proteins to the Nucleolus, in Mammalian Cells

**DOI:** 10.1371/journal.pone.0059065

**Published:** 2013-03-13

**Authors:** Ivan S. de Melo, Maria D. Jimenez-Nuñez, Concepción Iglesias, Antonio Campos-Caro, David Moreno-Sanchez, Felix A. Ruiz, Jorge Bolívar

**Affiliations:** 1 Departamento de Biomedicina, Biotecnología y Salud Pública - Facultad de Ciencias, Universidad de Cádiz, Cádiz, Spain; 2 Unidad de Investigación, Hospital Universitario Puerta del Mar, Cádiz, Spain; 3 Departamento de Biomedicina, Biotecnología y Salud Pública - Facultad de Medicina, Universidad de Cádiz, Cádiz, Spain; Queen’s University, Canada

## Abstract

NOA36/ZNF330 is an evolutionarily well-preserved protein present in the nucleolus and mitochondria of mammalian cells. We have previously reported that the pro-apoptotic activity of this protein is mediated by a characteristic cysteine-rich domain. We now demonstrate that the nucleolar localization of NOA36 is due to a highly-conserved nucleolar localization signal (NoLS) present in residues 1–33. This NoLS is a sequence containing three clusters of two or three basic amino acids. We fused the amino terminal of NOA36 to eGFP in order to characterize this putative NoLS. We show that a cluster of three lysine residues at positions 3 to 5 within this sequence is critical for the nucleolar localization. We also demonstrate that the sequence as found in human is capable of directing eGFP to the nucleolus in several mammal, fish and insect cells. Moreover, this NoLS is capable of specifically directing the cytosolic yeast enzyme polyphosphatase to the target of the nucleolus of HeLa cells, wherein its enzymatic activity was detected. This NoLS could therefore serve as a very useful tool as a nucleolar marker and for directing particular proteins to the nucleolus in distant animal species.

## Introduction

The nucleolus is a dynamic structure that disassembles and reforms during each cell cycle around the rRNA gene clusters [1]. Nucleolus is a major nuclear substructure that coordinates many cellular activities including ribosomal production [2], cell cycle control [3], DNA damage repair [4] and tRNA processing [5]. In order to get into the nucleus, nucleolar proteins must be targeted to the nucleus through the nuclear pore complex (NPC) [6]. Small molecules can move freely from the cytoplasm through the NPC, but transport of proteins larger than 40 kDa is mediated by specific amino acid sequences, referred as nuclear localization signal (NLS) [7], [8]. A classic NLS contains a cluster of basic amino acids, typically composed of lysines (K) or arginines (R), organized in either a single stretch, the monopartite NLS ((K/R)4–6), or in two stretches, the bipartite NLS, where two small clusters are separated by a few amino acids ((K/R)2X10-12(K/R)3) [9]. Unlike the nucleus, the nucleolus is a membrane-free nuclear structure. Nucleolar localization of proteins is mediated typically by functional interaction with nucleolar core components, such as ribosomal DNA, RNA and proteins, yet in most of the cases depends on nucleolar localization sequences (NoLSs) [10]. These kinds of signals have been found in cellular and in viral nucleolar proteins [11]. NoLSs and NLSs have very similar amino acid compositions (a high prevalence of basic residues in both cases) and in some proteins the same region can both target proteins across the nuclear envelope and cause proteins to accumulate in the nucleolus (for example, UTP20 is reported to contain overlapping NLS and NoLS near its C-terminus) [12]. However, in others proteins the NoLS cause proteins to accumulate in the nucleolus but are unable to mediate nuclear envelop translocation. In these cases the proteins usually also contain a NLS (for instance, PPP1R11 contain two different signals: a NoLS-only signal and a NLS-only signal [8]. Because the nucleolus is not a membrane-bound structure, it is presumed that nucleolar accumulation occurs via interaction with already-established nucleolar components. Therefore, members of a subset of the nucleolar proteome act as building blocks of the nucleolus around rDNA genes, and it is formed in an incremental manner [13]. Consequently, many nucleolar proteins may accumulate in a steady-state compartment mediated by NoLS (i.e. a sequence or domain) which might interact with local high-affinity binding sites [14].

A number of NoLSs have been described in several retroviral proteins and cellular proteins. Amino acid sequence analyses revealed that often contain clusters of basic amino acids, mainly the Arg-rich or Lis-rich region, that diverge in context and length [14], [15], however a consensus sequence is not generally found in cellular nucleolar proteins (Table 1).

**Table 1:** Sequences of several nucleolar localization signals in which one or more clusters of basic amino acids are present.

Protein name	NoLS	Ref.
MDM2	**KK**L**KKR**N**K**	[16]
P14ARF	**RR**GAQL**RR**P**R**HSHPT**R**A**RR**CP	[17]
SVV-EX3	MQ**RK**PTI**RRK**NL**RK**L**RRK**	[15]
NIK	**RKKRKKK**	[18]
hLa	QESLN**K**W**K**S**K**G**RR**F**K**G**K**G**K**GN**K**AAQPGSG**K**G**K**	[19]
ING-1	MTP**K**E**KK**A**K**TS**KKKKR**S**K**A**K**A	[20]
NOLP	**RKR**I**R**TYL**K**SC**RR**M**KRS**GFEMS**R**PIPSHLT	[21]
IGF-I	GTEASLQI**R**G**KKK**EQ**RR**EIGS**R**NAEC**R**G**KK**G**K**	[22]
Werner	VS**R**YN**K**FM**K**ICALT**KK**G**R**NWLH**K**ANTES	[23]
Nucleolin	**R**GGGGGGGDF**K**PQG**KK**T**K**F**R**	[24]
Parafibromin	**RR**AATENIPVV**RR**PD**RK-(X)_301_-KK**QGCQ**R**ENETLIQ**RRK**	[25]
PTHrP	G**KKKK**G**K**PG**KRR**EQE**KKKRR**T	[26]
DEDD	**KR**PA**R**G**R**ATLGSQP**KRRK**SV	[27]
Angiogenin	IM**RRR**GL	[28]
Protein Alpha	**RRR**ANN**RRR**	[29]
MEQ	**RRRKR**N**R**DAA**RRRRRK**Q	[30]
Tat	G**RKKRR**Q**RR**AP	[31]
Rev	**R**QA**RR**N**RRRR**WE**R**Q**R**	[32]
Rex	P**K**T**RRR**P**RR**SQ**RKR**P	[33]
I14L	MS**RR**N**KR**S**RRRRKK**PLNTIQ	[34]

These clusters of basic amino acids can be found in human proteins as MDM2 [16], p14ARF [17], SVV-EX3 [15], NIK [18], hLa [19], ING-I [20], NOLP [21], IGF-I [22], Werner [23], Nucleolin [24], Parafibromin [25], PTHrP [26], DEDD [27], Angiogenin [28] and in several viral proteins such as Protein alpha [29], MEQ (protein of Marek’s disease virus (MDV) [30], Tat [31] and Rev [32] (both proteins of human immunodeficiency virus type 1), Rex (protein of human T lymphotropic virus type [33], I14L (protein of African swine fever virus) [34]. Basic amino acids are shown in bold letters. Motifs of two or more K/R are underlined.

NOA36 is a protein that was described by us when studying a nucleolar autoantigen that was immunologically characterized in a patient suffering from rheumatoid arthritis with high levels of antibodies to the nucleolus organizer regions [35]. This protein is widely distributed in human tissues, with significantly higher expression levels in heart and skeletal muscle [35]. In addition, NOA36 has been highly conserved during evolution, and is present in many animals, from cnidarians to humans [35]. NOA36 is localized in the nucleoli and the mitochondria of mammalian cells [35]–[36]. We have recently demonstrated that mitochondrial NOA36 is released to the cytosol during apoptosis and becomes part of the cellular apoptotic machinery [36]. Despite the fact that a significant fraction of NOA36 has nucleolar localization, it still remains to be determined how the protein is delivered to and incorporated into the nucleolus. Localization of NOA36 in the nucleolus is RNA-dependent, since treatment of CHO cells with RNAses causes no nucleolar distribution, while treatment with DNAses does not change this localization [37]. Interestingly, NOA36 has been identified as a tyrosine kinase target in Jurkat cells [38].

Three different domains can be deduced in the NOA36 protein: a poly-acidic region at the carboxy-terminus, a long cysteine-rich domain, and an amino-terminal with a high proportion of basic amino acids. In this work, we demonstrate that the NoLS of NOA36 is located at the astonishingly well-conserved N-terminus of the protein, and that a sequence of three residues of the basic amino acid lysine is critical for its nucleolar localization. In addition, we also found that this peptide is able to target the functional heterologous expression of cytosolic yeast exopolyphosphatase scPPX1 inside the nucleolus of mammalian cells. This enzyme degrades specifically inorganic polyphosphate, a compound that has been recently associated with the nucleolar transcription [39].

## Materials and Methods

### Cell lines, Culture Conditions and Transfections

HeLa, CHO and PC3 cells (from ATCC) were cultured in Dulbecco’s modified Eagle’s medium at 37°C in a CO2 incubator; CHSE cells [40] were cultured in L-15 at 20°C; and S-2 (from Invitrogen) were cultured in Schneider’s medium at 25°C. All the cell culture media were supplemented with 10% foetal bovine serum and 100 U/ml penicillin and 100 µg/ml streptomycin. Most of the transient transfections were performed using FuGene HD, and 1.8 µg of plasmid in 35 mm plates, according to the manufacturer’s instructions. For transfections in HeLa cells with the vector pIRES 2eGFP, Lipofectamine LTX (Invitrogen) was used, according to the manufacturer’s instructions.

### Immunostaining

For indirect immunofluorescence staining, cells grown on coverslips were washed with PBS and fixed in cold acetone for 10 min at −20°C (anti-NOA36, anti-UBF and anti-Flag antibodies) or 4% paraformaldehyde before permeabilization with 0.1% Triton X-100 (eGFP transfected cells). Cells were then washed with PBS and incubated with primary antibodies diluted in PBS (1∶100 of anti-NOA36; 1∶100 of anti-UBF or 1∶500 of anti-Flag M2 (SIGMA)) at 37°C for 45 min. Cells were then washed with PBS for 30 min at room temperature and incubated with Alexa fluor 488, 555 (Molecular Probes), or Cy3 (Jackson ImmunoResearch)-labelled secondary antibodies at 37°C for 45 min. Finally, cells were washed twice in PBS and mounted in PBS-glycerol containing DAPI at 0.1 µg/ml, for DNA staining. A Zeiss Axiophot microscope equipped with a 63×NA 1.3 oil-immersion objective was routinely used. Images were taken using a SPOT Camera (Diagnostic Instruments Inc.) and processed using Adobe Photoshop software.

### Cloning and Site Directed Mutagenesis

Mammalian expression cytomegalovirus promoter-based vector constructs encoding N-terminal tagged Flag-NOA36 and truncated Flag-N34-NOA36 (Invitrogen), C-terminal tagged NOA36-eGFP (Clontech) were generated by PCR amplification from the human full-length NOA36 cDNA. PCR products incorporated EcoRI and the BamHI sites on the products ends. A truncated fusion protein containing amino acids 1–33 of NOA36 was also expressed as eGFP fusion protein. Site-directed mutagenesis was carried out on this p1-33-NOA-eGFP truncated protein to generate deletion of several amino acids, or alternatively, to convert a basic amino acid to a neutral amino acid. The sequence data for the mutagenic oligonucleotides are shown in Table S1. The entire coding region of the yeast exopolyphosphatase, scPPX1, was obtained from the plasmid pTrcPPX1 [41], which was kindly provided by the late Prof. Arthur Kornberg, Stanford University School of Medicine (Stanford, CA). Coding sequences for scPPX1 and for the residues 1–33 of NOA36 were ligated to a vector pcDNA3.1 (Invitrogen) that had previously been modified to contain a Flag epitope. The sequence for the resultant recombinant protein, Flag-1-33-NOA36-scPPX1, was further subcloned into the eukaryotic expression vector pIRES2-EGFP (Clontech, Mountainview, CA). As a control, the coding sequence of scPPX1 was fused directly with the Flag tag and cloned into the eukaryotic expression vector pIRES2-EGFP. Constructs were verified by sequencing (Servicio Central de Ciencia y Tecnología, University of Cadiz). Sequences of oligonucleotides and mutagenesis primers are available on request.

### Nucleolar Localization Analysis

Leica confocal software (LCS Lite) was used to quantify the numbers of nucleolar and nuclear (excluding nucleoli) eGFP fusion proteins in fixed transfected HeLa cells. All the images (1024×768 pixels) were obtained using a TCM-SL confocal microscope (LEICA) with a 63× objective lens. Confocal images were quantified using settings where the intensity of eGFP fluorescence in the analyzed transfectans was linear and ranged from 10 to 160 pixel values [42]. Usually, 10 sections encompassing an entire cell were taken at 1.0-µm interval. To examine the localization of eGFP and eGFP fusion proteins, a square with an area of 50 pixels was used to measure the mean intensities of three different regions in two compartments - the nucleolus and the nucleus (excluding the nucleolus) - of four representative cells from each of three transfections. Thus, the twelve representative cells yielded a total of 36 areas from the nucleolus and 36 areas from the nucleus. Relative nucleolar and nuclear localization is reported as the mean of the percentage of the relative intensity of GFP fluorescence within a cell.

### Localization of Intracellular Polyphosphate and Flag Epitope

Co-detection of the intracellular localization of polyphosphate and the Flag epitope was performed using a laser TCS-SL confocal imaging system (Leica Microsystems). The Flag epitope was immunostained as described above, using mouse anti-Flag and Cy3-labeled secondary antibodies. Intracellular polyphosphate was detected as described before [39], [43], [44]; briefly, fixed and washed cells were incubated with 1 mg/ml of DAPI, mounted on slides, and specifically labelled for polyphosphate (excitation: 458 nm, emission: 530–570 nm).

### Cellular Protein Extraction and Western Blot Analysis

For cellular protein extraction, 1×10^6^ transfected HeLa cells were washed with PBS, and cell pellets were resuspended in 0.5 ml of lysis buffer (50 mM Tris-Cl, pH 7.5, 1% NP-40, 1% Na-deoxycholate, 0.1% SDS, 2 mM EDTA, 0.5 M NaCl, and 1/500 of SIGMA-protease inhibitor cocktail for mammalian cells). Preparations were incubated for 5 min in ice and then were passed through a narrow gauge needle (21G) to shear DNA. Then the lysates were cleared by centrifugation (14.000 g, 10 min at 4°C). Supernatants were used as fractions with the extracted proteins. MicroBCA Protein Assay Kit (Pierce) was used to determine protein concentrations. Western blot analysis was performed as described [36], but using 60 µg aliquots of extracted proteins and specific anti-Flag (SIGMA) or anti-eGFP (Abcam) primary antibodies.

### Statistics

Comparisons between groups were performed with Student’s T-test for paired samples (two-tailed) (n = 12) and significant differences (p<0.05) are denoted by an asterisk.

## Results

### 3.1. NOA36 Fused to Flag Epitope is Localized to the Nucleolus

**Figure 1:**
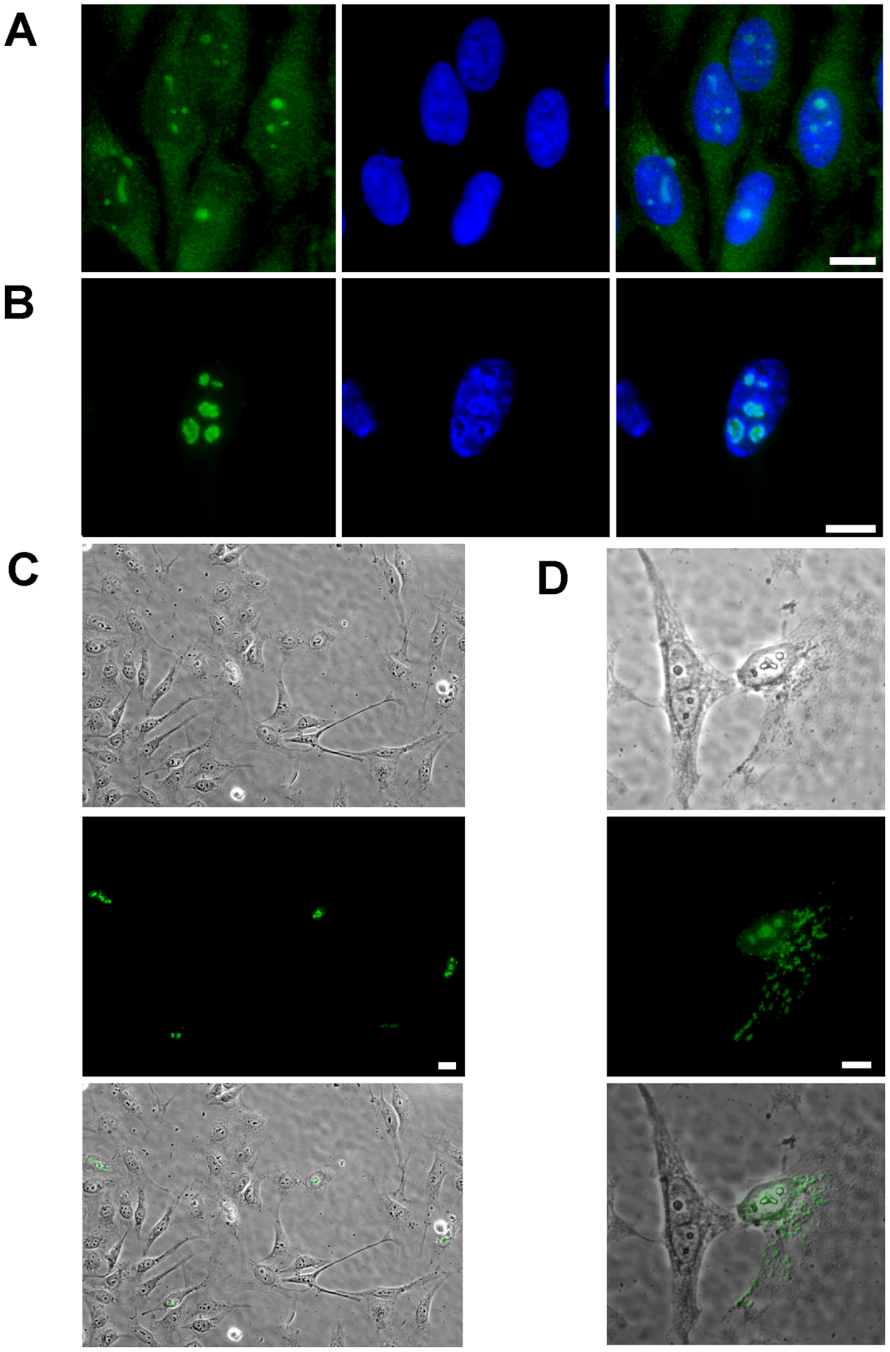
NOA36 is located in the nucleoli of mammalian cells. A) Indirect immunofluorescence labelling of HeLa cells with an anti-NOA36 polyclonal antibody (in green). B), C) and D) Transfection assays with the construct p-Flag-NOA36 in HeLa cells (labelled in green with an anti-Flag monoclonal antibody). In A) and B) chromatin localization (blue) and merged images are also shown in all the assays. In C) an D), images taken with differential interference contrast (upper panel), along with the anti-Flag staining (in green, central panel). A merge image of both pictures has been added to facilitate a better estimation of the recombinant Flag-NOA36 distribution in cell population (lower panel). C) Several transfected cells in which the Flag-NOA36 nucleolar distribution (in green, central panel) can be evaluated. D) Flag-NOA36 can also be found at cytoplasm of 12.5% either as a diffuse staining or in a punctuated pattern showed in D. Bars: 10 µm.

**Figure 2:**
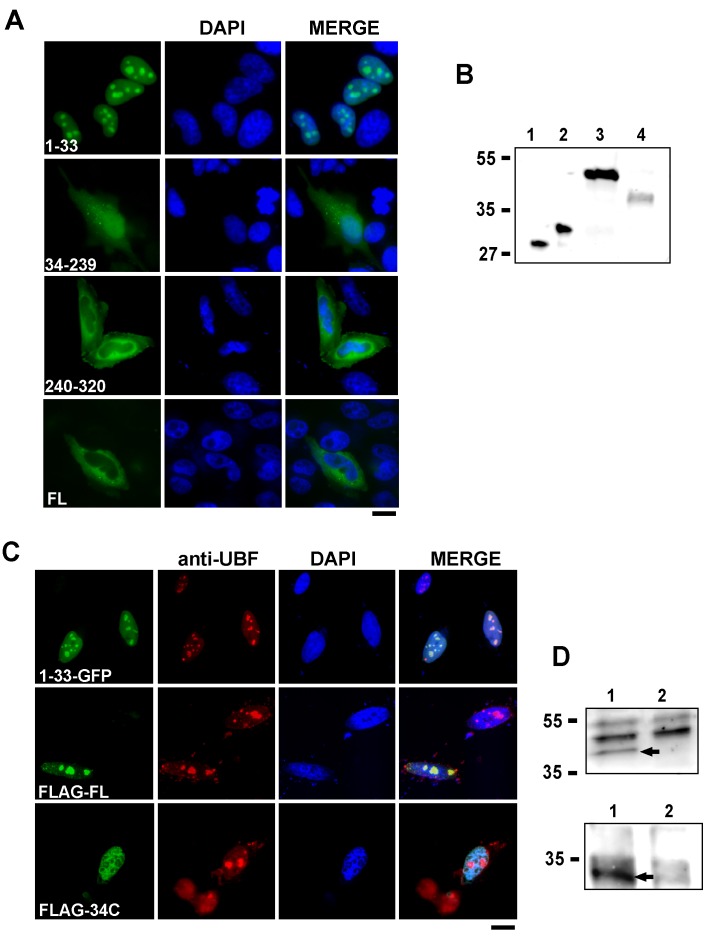
The NLoS of NOA36 is located at the amino terminal sequence. A) Transient transfection assays in HeLa cells with constructs expressing truncated peptides from the NOA36 human sequence fused to eGFP. The different peptides correspond to the three putative domains deduced from the NOA36 amino acid sequence: the amino terminal basic-rich peptide (construct 1–33), the central cysteine-rich domain (construct 34–239) and the polyacidic carboxy terminal sequence (construct 240–320). The cytoplasmic localization of the full-length protein fused to eGFP is also shown. B) Western blot analysis with an anti-eGFP antibody of protein extracts of HeLa cells transfected with the eGFP empty vector and with the different truncated fusion proteins showed in A). Line 1 eGFP; line 2, construct 1–33, line 3, construct 34–239; line 4, construct 240–320. Bars on the left indicate molecular weight markers. C) Transient transfection assays in HeLa cells with different constructs that demonstrate that the NoLS of NOA36 is included in the amino terminal sequence (amino acids 1–33) of the protein. To determine the localization of the nucleolus in the transfected cells, an indirect immunofluorescence assay with an antibody against the nucleolar marker UBF (in red) was performed in all the transfection experiments. In green cells transfected with the construct 1-33-eGFP (upper panel), the full length NOA36 human sequence fused to the carboxy terminus of the Flag epitope (construct Flag-FL, central panel), and a truncated NOA36 protein lacking the amino acids 2 to 33 fused to the Flag epitope (construct Flag-34C, lower panel). Chromatin staining with DAPI and merged images are also shown in (A) and (C). Bars: 10 µm. D) Western blot analysis with an anti-Flag antibody of protein extracts of HeLa cells transfected with the construct Flag-FL (upper panel, line 1), the construct Flag-34C (lower panel, line 1), and the p-Flag empty vector (line 2 in both panels panel). Bars on the left indicate molecular weight markers. The specific signals are indicated in both panels with an arrow.

We studied NOA36 subcellular localization using the Flag epitope as a reporter tag, with the aim of reproducing the nucleolar localization of the endogenous NOA36 (Fig. 1 A). For this, the Flag epitope fused to the amino terminal of NOA36 was localized at discrete points in the nucleus of transiently HeLa transfected cells, (Fig. 1 B and 1C) in 100% of the interphase cells, although in a 12.5% or the transfectants, a diffuse or punctuated cytoplasmic pattern could also be appreciated (Fig. 1D). The nucleolar localization of Flag-NOA36 recombinant protein was confirmed by using an anti-UBF antibody as nucleolar marker [45] (Fig. 2C). The nucleolar staining was not homogeneous, being localized mainly on the periphery of the nucleolus; this localization was more obvious in the larger nucleoli. From the data previously reported we concluded that NOA36 contains at least one nucleolar localization signal.

### 3.2. The NoLS of NOA36 is Localized at its N Terminus

To investigate the localization of the different regions of the NOA36 protein, we fused eGFP to three truncated peptides corresponding to the different putative domains deduced from the amino acid sequence: the amino terminal domain which extends from the first methionine to the last conserved basic amino acid before the cysteine-rich domain (amino acids 1 to 33); the central cysteine-rich region (amino acids 34 to 239); and the polyacidic carboxy terminal (amino acids 240 to 320). These constructs were analyzed in transient transfection assays in HeLa cells to determine the subcellular localization of the fusion proteins. Out of the three constructs tested, only the basic rich amino terminal peptide was capable of transporting eGFP to the nucleolus (Fig. 2). The cysteine-rich domain was distributed all over the cell, and the polyacidic carboxy terminal showed only a cytoplasmic localization. This cytoplasmic localization was similar to that found in the assays with the full-length protein fused to eGFP (Fig. 2A). To confirm that it is the amino terminal that is responsible for NOA36 nucleolar localization, we fused the Flag epitope to the amino terminus of a NOA36 truncated protein in which the amino acids 2 to 33 had been deleted. HeLa cells transiently transfected with this construct showed nuclear but not nucleolar staining of the Flag epitope, which was verified by the absence of colocalization with the nucleolar marker UBF in all the interphase transfected cells (Fig. 2C). Besides the nuclear staining, the 13.6% of the transfectants, also showed a diffuse or punctuated cytoplasmic pattern similar to that observed in the Flag-NOA36 fusion protein. These results demonstrated that the amino terminal sequence of NOA36 is the determinant for its nucleolar localization. Western blots analysis of the different truncated fusion proteins with an anti-eGFP antibody (Fig. 2B) or an anti-Flag antibody (Fig. 2D) confirmed that the full length proteins were expressed (Fig. 2B and 2D).

### 3.3. NOA36 Contains a Highly Evolutionary-preserved NoLS

**Figure 3:**
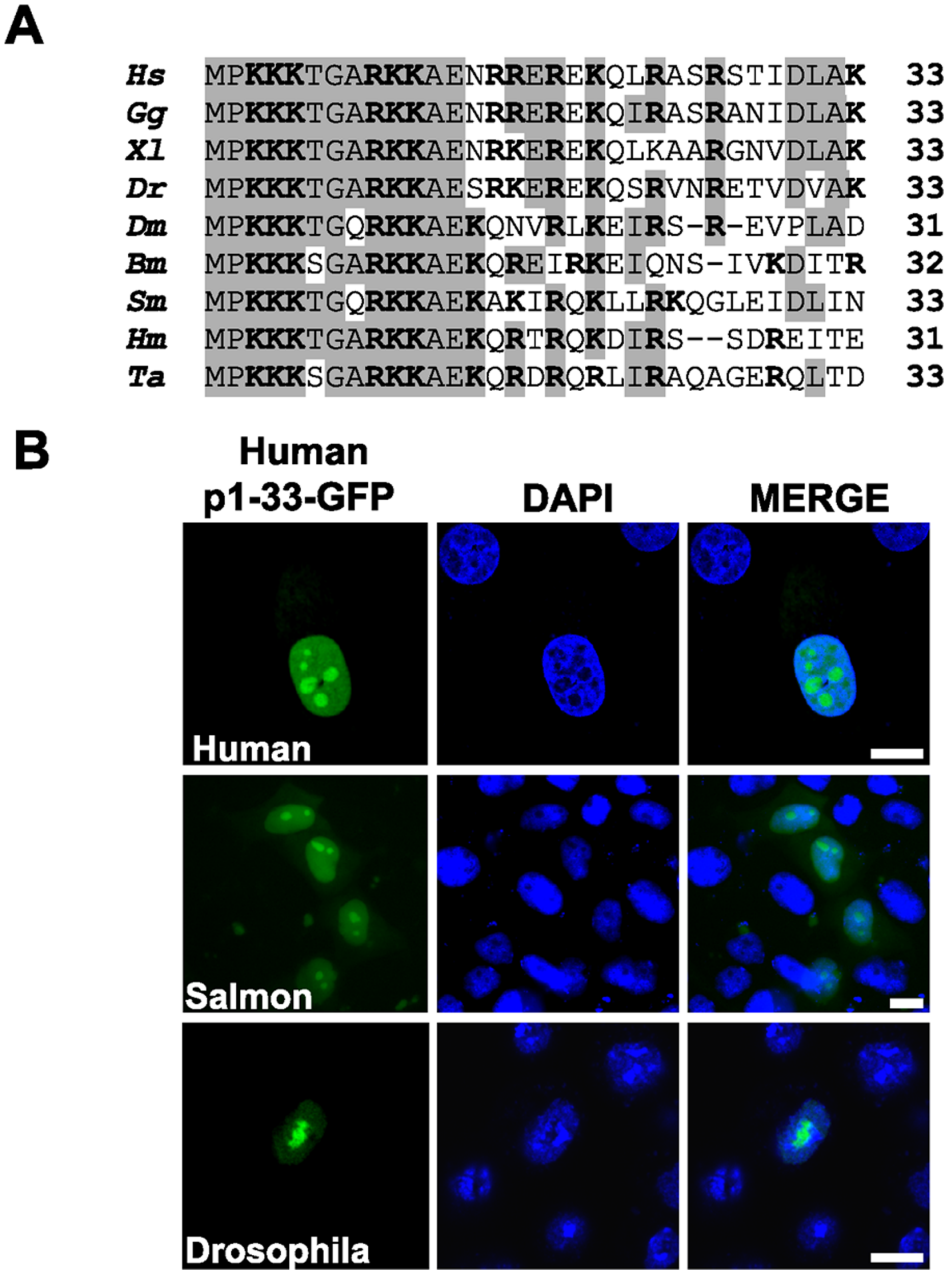
The NLoS of NOA36 is a highly evolutionary preserved motif. A) Clustalw alignment of the NOA36 amino terminal sequence from human (CAC18679.1) (Hs), Chicken (XP_001232423.1) (Gg), *Xenopus laevis* (ABF71466.1) (Xl), zebra fish (AAH46062.1) (Dr), *Drosophila melanogaster* (AAF56799.1) (Dm), the filarid worm *Brugia malayi* (CAD22104.1) (Bm), the flatworm Schistosoma mansoni (XP_002579681) (Sm), the cnidarian *Hydra magnipapillata* (XP_002155133) (Hm) and the placozoa *Trichoplax adherens* (XP_002110470) (Ta). Identity within the amino acid sequence in more than five orthologous sequences is highlighted by shaded boxes. Basic amino acids arginine (R) and lysine (K) are indicated in bold. B) Transient transfection assays with the human construct p1-33-eGFP in PC3 human cells, CHSE salmon cells and S-2 *Drosophila* cells. DAPI staining of the chromatin and merged images are also shown. Bars: 10 µm.

The human NOA36 amino terminal sequence contains a high proportion of lysine and arginine residues, with basic amino acids accounting for 40% in the sequence 1 to 33. The analysis of this sequence with Prosite software predicted the existence of a putative bipartite nuclear localization signal (NLS) between amino acids 3–20 [35]. The alignment of the NLS of human NOA36 with other NOA36 sequences from phyla as distant from each other as cnidarians and mammals reveals a remarkable degree of conservation, showing an identity of 66.6% (Fig. 3A). This peptide contains two clusters of three basic amino acids as well as other residues conserved in all the analyzed NOA36 protein sequences. The consensus sequence in the 20 first amino acids is **M**-**P**-**K**-**K**-**K**-S/T-**G**-A/Q-**R**-**K**-**K**-**A**-**E**-N/S/K-R/Q-R/K/N-E/V-R/I-X-**K**. However, other basic amino acids are present in the sequence 21 to 33 that could be significant for the nucleolar localization. In order to characterize the ability of the NOA36 amino terminal sequence to direct eGFP to the nucleolus in different species, we studied the localization of the recombinant protein 1–33NOA-eGFP by fluorescence microscopy in transient transfected cell cultures from different cell types and species. Our results showed that this peptide is sufficient to direct eGFP to the nucleolus in all the interphase cells in two different human cell lines, HeLa (fig. 2C) and PC3 (Fig. 3B), and in hamster CHO cells (data not shown). We found that this NoLS has been highly conserved during evolution, since fish and insect cells transfected with this construct also show nucleolar staining (Fig. 3B). These results are consistent with the high degree of conservation presented by this peptide over the course of evolution.

### 3.4 Three Lysine Amino Acids in Positions 3 to 5 are Critical for the NoLs of NOA36

**Figure 4:**
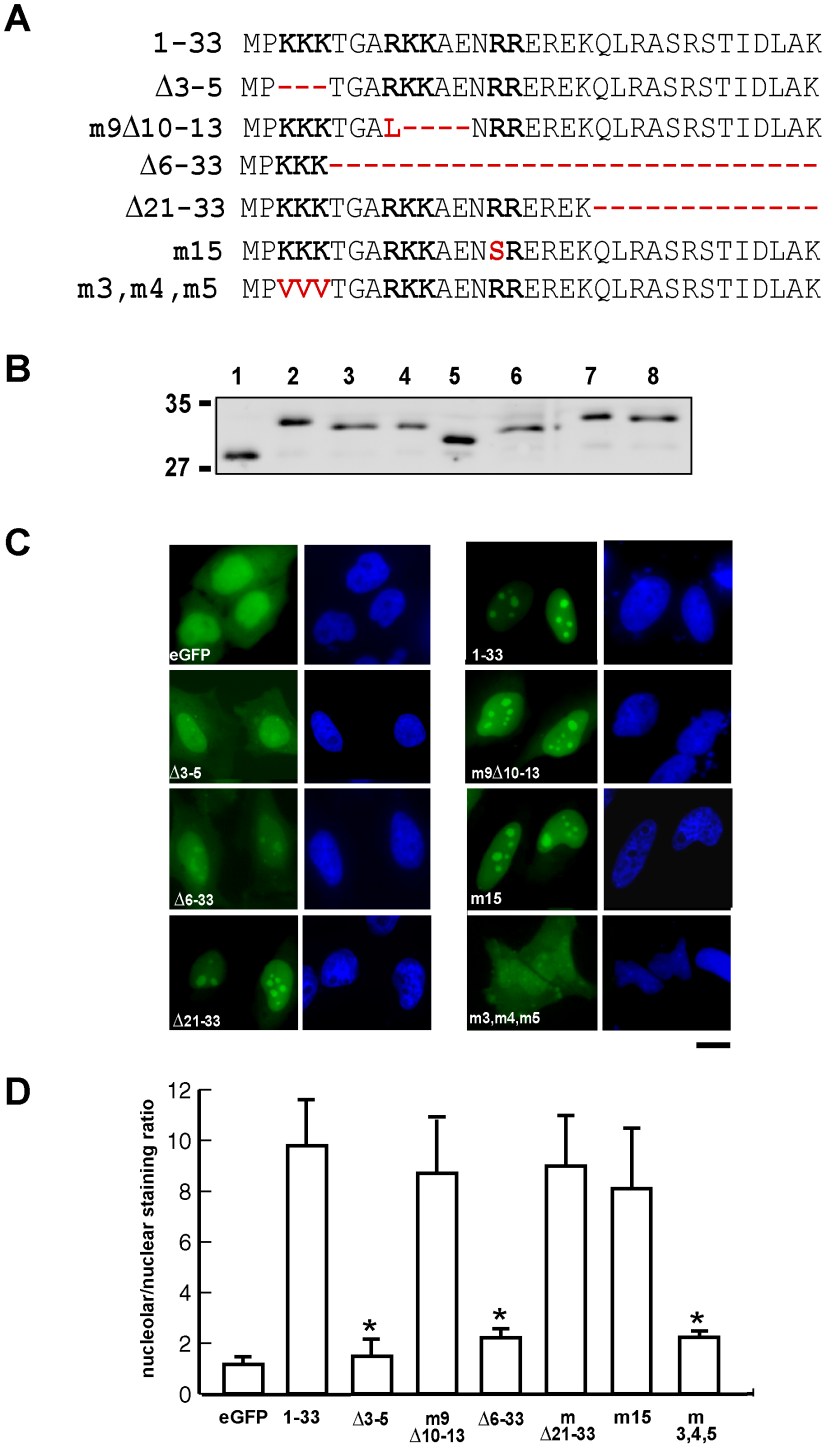
Amino acids 3 to 5 are critical for the NoLS of NOA36. A) Sequence of amino acids 1 to 33 of the NoLS of NOA36 in human, and the sequences of the different constructs in which this wild type sequence was mutated. The changes with respect to the wild type construct are marked in red. The clusters of two or three basic amino acids are highlighted in bold. B) Western blot analysis of protein extracts of HeLa cells transfected with the eGFP empty vector and with the different mutant proteins showed in A). Line 1, eGFP empty vector; line 2, construct 1–33; line 3, construct Δ3–5; line 4, construct m9Δ10–13; line 5, construct Δ6–33; line 6, construct Δ21–33; line 7, construct m15; line 8, construct m3,m4,m5. Bars on the left indicate molecular weight markers. C) Fluorescence microscopy analysis showing the eGFP distribution in HeLa cells transiently transfected with the eGFP empty vector and the constructs described in (A). D) Diagram showing the quantification of the relative eGFP staining within the cell (nucleolar/nuclear staining ratio), of the eGFP empty vector and the different constructs described in (A). Confocal images from three different experiments were analysed using “LCS Lite” software from Leica. Each bar represents the average ratio of nucleolus/nucleus eGFP intensity. Error bars represent standard deviations. Significant differences (*P<*0.05) with respect to the wild type sequence (1–33) are marked by asterisks (*).Bar: 10 µm.

We investigated which are the key elements of the NoLS of NOA36. To this end, we used the construct p1-33-eGFP as a reference and then we altered the protein by deleting several residues or changing basic amino acids to neutral amino acids (Fig. 4A). These modifications were made in order to eliminate one or more of the three clusters present in this peptide of two or three basic amino acids (**K-K-K**-T-G-A-**R-K-K**-A-E-N-**R-R**), which is a frequent feature in the nucleolar localization signals identified in other proteins [46], [47]. These constructs, together with the eGFP empty vector were transiently transfected in HeLa cells, and the eGFP localization was visualized by fluorescence microscopy (Fig. 4C). Western blots analysis of the different mutants proteins with an anti-eGFP antibody confirmed that the full length proteins were expressed (Fig. 4B). These transfectants were also analyzed by confocal microscopy in order to quantify the eGFP intensity in the nucleolus and in the nucleus (excluding the nucleolus) (Fig. 4D). This quantification revealed that the wild-type peptide (1–33) showed a nucleolus/nucleus ratio of 9.8, versus a ratio of 1.1 in the eGFP empty vector.

The three clusters of basic amino acids of this NoLS are highly conserved during evolution, since the first two clusters are present in all the available orthologous sequences of NOA36, from cnidarian to mammalian species, while the third cluster is present in all the vertebrate sequences analyzed (Fig. 3A). We therefore decided to study the effect of deleting each of these clusters in turn (constructs (Δ3–5) and (m9Δ10–13)). The analysis of the quantification of these constructs revealed that only the cluster of three lysine residues in positions 3–5 was essential for maintaining the NoLS, since the deletion of these three lysine residues provoked a nuclear distribution of eGFP very similar to that of the empty vector (nucleolus/nucleus ratio = 1.5). This effect was confirmed by a mutant in which these three basic amino acids were changed to neutral amino acids (nucleolus/nucleus ratio = 2.2). The elimination of the second cluster (amino acids 9–11), however, did not show statistically significant differences in fluorescence intensity with respect to the wild type peptide, although it did show a lower nucleolus/nucleus ratio (8.7). Similar results were obtained when an Arg residue was mutated to Ser (m15) in the third cluster (nucleolus/nucleus ratio = 8.1). These data suggest that the main role in the nucleolar localization is played by the first cluster of lysine residues in position 3–5, with the other two clusters of basic amino acids playing a minor role. These data also suggest that the first cluster alone might be sufficient to promote nucleolar transport of the eGFP. In order to answer this question, that is, whether this first cluster is in fact the NoLS of NOA36, we fused amino acids 1–5 to eGFP (construct (Δ6–33)). However, the quantification of the relative fluorescence intensity of this construct revealed that, although the nucleolar localization was slightly higher than in the empty vector (nucleolus/nucleus ratio = 2.2), this short peptide did not retain the capability of transporting the eGFP to the nucleus (nucleus/cytoplasm ratio = 3) (Fig. 4C and D). We also studied the significance of the amino acids not included in the basic amino acids region (amino acids 21 to 33). With this aim, we eliminated this sequence from the reference peptide (construct (Δ21–33)). The quantification of fluorescence intensity revealed that there were no statistically significant differences when it was compared with the pN33-eGFP reference construct (nucleolus/nucleus ratio = 9) (Fig. 4C and 4D).

### 3.5 The NoLS of NOA36 Targets the Functional Heterologous Expression of Proteins in the Nucleolus

**Figure 5:**
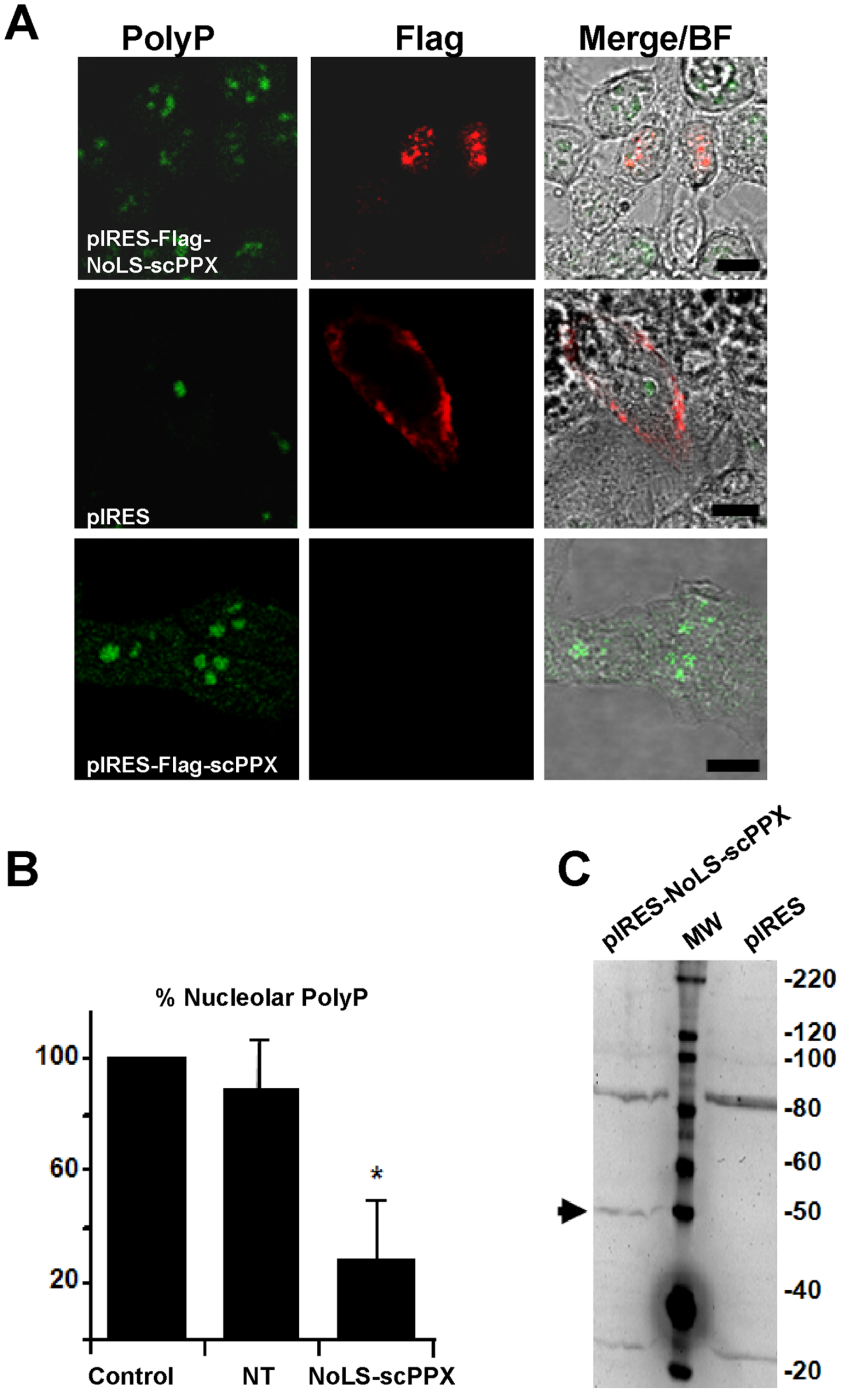
Targeted expression of polyphosphatase (scPPX) by the NoLS of NOA36 reduces levels of nucleolar polyphosphate in HeLa cells. A) Confocal fluorescence microscopy of fixed HeLa cells transfected with a vector with: the NoLS-scPPX construct (pIRES-NoLS-scPPX), a scPPX construct (pIRES-scPPX), or an empty vector (pIRES). Specific labelling of polyphosphate (PolyP, green) and Flag antibody (NoLS-scPPX, red) was determined. A representative experiment is shown (n = 3). Bar: 10 µm. B) Densitometric analysis of the polyphosphate signal at the nucleolus in control cells (control), and in cells after the transfection procedure with the NoLS-scPPX construct (NT and NoLS-scPPX). Co-labelling with Flag antibody allows the signals of non-transfected cells (NT) and positive transfected cells (NoLS-scPPX) to be separated. Analysis was performed using ImageJ 1.43u software. Results represent the mean ± S.D. of the measurements in 150 cells in each condition (n = 3). Asterisk indicates significant differences in comparison with non transfected cells, determined by T-test (P<.002). C) Western blot expression of Flag-polyphosphatase protein in cell extracts of cells transfected with an empty vector (pIRES), or with a vector with the NoLS-scPPX construct (pIRES-NoLS-scPPX). Protein molecular weight markers are shown in the middle of the panel (MW), and their estimated protein sizes (in kDa) are indicated to the right. The expected molecular weight for the polyphosphatase protein (∼50 kDa) is indicated with an arrow. Nonspecific bands around 85 and 30 kDa appear for all analyzed samples. A representative experiment is shown (n = 3).

We constructed DNA encoding the NoLS of NOA36 fused with the Flag epitope and an exopolyphosphatase enzyme from yeast (scPPX1) that specifically hydrolyzes polyP into inorganic phosphate [41]. The expression of the protein in the nucleolus of transiently transfected cells was confirmed by confocal microscopy. Fig. 5A shows the immunolocalization of the Flag epitope, which was positive in at least 60% of the cells in all cases. Positive transfected cells with NoLS-scPPX showed a drastic reduction (less than 30% of the control) of the polyphosphate signal in the nucleolus (Fig. 5A, B). In addition, when lacks NoLS, the positive transfected cells with scPPX show non-nucleolar localization at the same time as high amounts of nucleolar polyphosphate (Fig. 5A). We confirmed the expression of the fused protein in extracts of transfected cells by western blot of the Flag epitope (Fig. 5C). Only the cells transfected with the construct that included the fused protein showed a positive signal for the Flag epitope (Fig. 5C).

## Discussion

In recent years, the nucleolus has emerged as a complex structure implicated in important processes beyond its key role in ribosomal biogenesis, a point of view supported by recent proteomic analysis [48]. Although we have previously described the pro-apoptotic role of NOA36 in the mitochondrial apoptotic pathway, the role played by this protein in the nucleolus has not yet been elucidated. As a basis for this investigation, in this work we have identified the specific amino acid sequence responsible for transporting NOA36 into the nucleolus. This subnuclear structure, the nucleolus, is reformed during each cell cycle and, because it is not isolated by a membrane, proteins do not need a specific transport mechanism in order to penetrate this compartment. Although some efforts have been made in order to characterize and classify NoLs [8], the signal that gives rise to this trans-localization into the nucleolus is difficult to predict and is sometimes contained within or overlapped by the nuclear localization signal [12]. Proteins seem to accumulate in the nucleolus in a steady-state manner and, in this view, there are different mechanisms by which proteins are retained in the nucleolus: there are proteins that bind to rDNA (i.e. with DNA binding motifs) [49] or to rRNA (i.e. containing RNA binding motifs) [19]. Other proteins are anchored to the nucleolus by binding to one of those mentioned above. Some of these proteins (hub proteins) are able to interact with several others that incorporate in their sequence one or more NoLSs, and these signals are characteristically rich in basic amino acids [50], [51]. Finally, other proteins may be located in this subnuclear structure by interacting with other nucleolar proteins in a non-predictable manner. In this work, we have found that NOA36 is transported to and localized in the nucleolus as the result of a specific peptide signal, therefore this protein probably interacts with a RNA binding protein, since the nucleolar localization of NOA36 depends on RNA but not on DNA [37].

The NoLS of NOA36 is characterized by three clusters of basic amino acids that have been astonishingly well-conserved during evolution. We found that one of these clusters (amino acids 3 to 5) is critical for nucleolar localization, although this sequence is not sufficient to maintain the capability of nucleolar targeting. Therefore, we concluded that other basic amino acids located near this sequence are needed in order to enhance nucleolar accumulation even though their precise position with respect to this cluster does not seem to be important.

The high degree of homology presented by this NoLS in all the NOA36 orthologous sequences could suggest a very well-preserved mechanism for nucleolar retention, indicated not only by the similarity of the amino acid sequences between distant species, but also by the ability of the human sequence to transport eGFP to the nucleolus in fish and insect cells.

We also have demonstrated that the NoLS of NOA36 can be used to target the expression of yeast exopolyphophatase in the nucleolus of the HeLa cell. Polyphosphate is a ubiquitous polymer, formed by phosphate (Pi) residues, that directly regulates several processes in the cell and is used as a phosphate donor in gene regulation [52]. In mammalian cells, the enzymes involved in polyphosphate metabolism are still unknown; therefore, new tools to specifically modify subcellular levels of polyphosphate should be very useful. We recently have described the nucleolar localization of polyphosphate in eukaryotic cells, which is associated with nucleolar transcription by modulating the RNA Pol I activity [39]. As myeloma cancer cells have an exceptionally high level of polyphosphate in the nucleolus [39], the NOA36 NoLS-scPPX construction could be of special interest for prospective studies to understand the function of nucleolar polyphosphate on the biology of myeloma cells.

An important aspect in the characterization of NOA36 NoLS is to clarify whether this sequence is only a NoLS-signal, or would act both as a NoLS and NLS signal. In this sense our data are somehow contradictory: on the one hand, the fusion of NOA36 NoLS to the yeast exopolyphophatase is sufficient to transport this cytoplasmic protein to the nucleoli of HeLa cells (Flag-NoLS-scPPX1 construct, 50.4 kDa, Fig. 5A). This would indicate that the amino terminal sequence of NOA36 could be a joint NoLS-NLS region. On the other hand, the full length NOA36-eGFP fusion protein (63.7 kDa) normally localizes in the cytoplasm of HeLa and CHO cells, although shows also nucleolar localization in a small fraction (less than 1%) of CHO transfected cells [35]. This fact could indicate that this sequence would not be sufficient to transport a protein larger than the nuclear pore exclusion size (<40 kDa). Because of its molecular weight (36 kDa), NOA36 should not need an active transport facilitated by specific soluble carrier proteins in order to get into the nucleus.

Nevertheless, intracellular localization of endogenous NOA36 might be a more complex issue since not only is it located in the nucleolus; but it is also in the mitochondria [36]. In this sense we found that, besides the NoLS, NOA36 could contain a cytoplasmic retention signal located at the carboxy terminus. This fact can be deduced from the localization of the truncated protein 240–310 fused to eGFP. This 36.9 kDa fusion protein should passively diffuse across the nuclear pore complex and enter the nucleus. However, it does not seem to accumulate in the nucleus and it shows mainly cytoplasmic localization (Fig. 2A). Cytoplasmic retention signals (CRS) or cytoplasmic localization signals (CLS) have been previously reported in several human proteins, like Cycline B2 [53], the HIV-1 Host Defense Factor APOBEC3G [54] and PTEN [55]. Nevertheless endogenous NOA36 is not a cytosolic, but a mitochondrial protein. However, the characterization of the signal responsible of NOA36 mitcochondrial localization remain elusive in our hands, basically, due to the fact the reporters we have used so far (eGFP, RFP, mCherry and Flag) seem to interfere with the mitochondrial localization of the protein. In the case of the Flag-NOA36 construct, the protein localizes at the nucleoli, although can be also detected in the cytoplasm of the 12.5% of transfected cells in a diffuse or punctuated pattern (Fig. 1D). On the other hand when the NoLS is removed from NOA36 (construct Flag-34C), the recombinant protein shows a nuclear but not nucleolar localization (Fig. 2C), which would suggest the presence of an additional nuclear localization signal within this truncated protein. Further investigations will be required to clarify the role of additional intracellular transport signals in NOA36.

Other signals for nucleolar localization have been described in various different proteins, but because NOA36 is present in animals, from cnidarians to mammals, it is probable that the NoLS of NOA36 could serve as a very valuable tool in many kinds of animal cell for transporting target proteins to the nucleolus, or as a nucleolar marker when fused to a fluorescent protein.

## Supporting Information

Table S1 **Sequence data for the oligonucleotides used in the mutagenesis.** The oligonucleotides contained the enzymes restriction sites (highlighted in red) which allowed the cloning of the PCR products into the peGPFN1 vector. Full length NOA36 cDNA was used as template for PCRs. For the m9Δ10−13 and the m14 constructs, two PCR products were generated, which were digested with the appropriate restriction enzymes and then ligated into the peGFPN1 vector in a two inserts and one vector reaction. The construct Δ6–33 was generated by annealing the forward and reverse oligonucleotides. The product of the annealing included protruding ends for EcoRI in 5′ end and BamHI for 3′ end.
(PDF)
