# Supplementary material for: NOA36 Protein Contains a Highly Conserved Nucleolar Localization Signal Capable of Directing Functional Proteins to the Nucleolus, in Mammalian Cells
Source: PLoS One. 2013 Mar 13;8(3):e59065. doi: 10.1371/journal.pone.0059065 (PMC3596294; doi:10.1371/journal.pone.0059065)
Supplement: Table S1 — Sequence data for the oligonucleotides used in the mutagenesis. The oligonucleotides contained the enzymes restriction sites (highlighted in red) which allowed the cloning of the PCR products into the peGPFN1 vector. Full length NOA36 cDNA was used as template for PCRs. For the m9Δ10−13 and the m14 constructs, two PCR products were generated, which were digested with the appropriate restriction enzymes and then ligated into the peGFPN1 vector in a two inserts and one vector reaction. The construct Δ6–33 was generated by annealing the forward and reverse oligonucleotides. The product of the annealing included protruding ends for EcoRI in 5′ end and BamHI for 3′ end. (PDF) [file pone.0059065.s001.pdf]

TABLE 1S

CONSTRUCT	FORWARD OLIGONUCLEOTIDE	REVERSE OLIGONUCLEOTIDE	PCR digestion	CLONING SITES IN pEGPN1
1-33	GGGGAATTCAGGGGAAAATGCCTAAAAAA	ATTACATGGATCCTTAGCTAAATC	EcoR I/BamH I	EcoR I/BamH I
Δ3-5	GGGGAATTCAGGGGAAAATGCCTACTGGTGCGAGGAAGAAG	ATTACATGGATCCTTAGCTAAATC	EcoR I/BamH I	EcoR I/BamH I
m9Δ10-13	GGGGAATTCAGGGGAAAATGCCTAAAAAA	CTTCAGCGCACCAGTCTTTTTTTTAGGC	EcoR I/BamH I	EcoR I/BamH I
	GAAGGCGCTGAACCGCCGAGAACGTG	ATTACATGGATCCTTAGCTAAATC	Hha I/BamH I	
Δ6-33	AATTCAGGGGAAAATGCCTAAAAAAAAG	GATCCTTTTTTTTAGGCATTTTCCCCTG	----	EcoRI/BamHI
Δ21-33	GGGGAATTCAGGGGAAAATGCCTAAAAAA	CCCCACCGGTTTTTCACGTTCTCGGCGG	EcoR I/Age I	EcoR I/Age I
m15	GGGGAATTCAGGGGAAAATGCCTAAAAAA	GTTTTTCACGTTCTCGGCTGTTCTCAG	EcoR I/Tail	EcoR I/BamH I
	CTGAGAACAGCCGAGAACGTGAAAAAC	ATTACATGGATCCTTAGCTAAATC	Tail/BamH I	
m3,m4,m5	GGGGAATTCAGGGGAAAATGCCTGTAGTAGTGACTGGTGC	ATTACATGGATCCTTAGCTAAATC	EcoR I/BamH I	EcoR I/BamH I
